# Avoidant/Restrictive food intake disorder in adults within the community: A mixed-method study on individual impairment and progression

**DOI:** 10.1186/s40337-026-01765-w

**Published:** 2026-09-10

**Authors:** Josefa Ilg, Anne-Katrin Merz, Lena Kramer, Alexander Nettlau, Anja Hilbert, Ricarda Schmidt

**Affiliations:** 1 Department of Psychosomatic Medicine and Psychotherapy, Behavioral Medicine Research Unit, Integrated Research and Treatment Center AdiposityDiseases, Leipzig University Medical Center, Leipzig (Saxony), Germany; 2 Department of Child and Adolescent Psychiatry, Psychotherapy and Psychosomatics, University Hospital Leipzig, Leipzig (Saxony), Germany

**Keywords:** Eating disorder, ARFID, Impairment, Progression, Lived experiences

## Abstract

**Background:**

While avoidant/restrictive food intake disorder (ARFID) occurs across the lifespan, most previous research focused on children and adolescents. Particularly, individual impairment and progression patterns in adults remain largely unknown. Therefore, this study aimed to explore individual impairment and to identify progression patterns and associated psychological and sociodemographic characteristics in adults with ARFID.

**Methods:**

Data from *N* = 97 adults from the community with an interview-based diagnosis of ARFID were utilized. Participants took part in an online study on ARFID and answered open-ended questions on personal impairment and disorder progression, which were analyzed based on a qualitative content-thematic approach. In addition, ARFID symptoms, obsessive-compulsive personality traits, temperament, and sensory sensitivity were assessed via established questionnaires. Quantitative analyses included comparisons of progression groups in sociodemographic and clinical variables.

**Results:**

Qualitatively, seven main themes of impairment emerged: (1) health-related impairment, (2) impairment of lifestyle, (3) impairment by the eating behavior itself, (4) impairment due to responses of the social environment, (5) partnership, (6) parenthood, and (7) impairment due to experiences with practitioners. Higher education and age were more often seen in individuals with improved compared to individuals with deteriorated progression patterns, while greater fear of aversive consequences was associated with deteriorated progression.

**Conclusion:**

This is the first study to comprehensively capture the impact of ARFID based on experienced impairments and to highlight the heterogeneity of disorder progression, with educational level, age, and ARFID symptoms emerging as potentially associated factors. Longitudinal studies are needed to prospectively identify risk and protective factors on ARFID’s progression and persistence.

**Supplementary information:**

The online version contains supplementary material available at https://doi.org/10.1186/s40337-026-01765-w.

## Abstract (plain language summary)

Avoidant/restrictive food intake disorder (ARFID) is an eating disorder that can affect both children and adults. However, most research has focused on children, while little is known about how adults experience the disorder in their daily lives and how their symptoms change over time. In an online study, 97 adults with a diagnosis of ARFID were asked to describe what they struggle with most due to their avoidant-restrictive eating behavior and how their symptoms have changed since onset. The results show that most adults with ARFID reported burden related to their health (68.8%), restrictions of everyday activities (39.6%), burden due to their eating behavior (34.4%), their social relationships (24.0%), including partnership (13.5%), family life (8.3%), and experiences with healthcare professionals (2.1%). In terms of symptom changes, four groups of adults with ARFID were found: improved symptoms (41.3%), worsened symptoms (18.5%), consistent symptoms (32.6%) or varying (7.6%) symptoms. Adults with an improvement of symptoms were older and more highly educated compared to those with worsened symptoms, whilst the fear to experience aversive consequences was higher in those reporting worsened symptoms. These findings help us better understand the challenges adults with ARFID face and may guide future support and treatment options.

**Supplementary information:**

The online version contains supplementary material available at https://doi.org/10.1186/s40337-026-01765-w.

## Background

Avoidant/restrictive food intake disorder (ARFID) is an eating and feeding disorder replacing and extending the former diagnosis of feeding disorder of infancy and early childhood in the Diagnostic and Statistical Manual of Mental Disorders [1, 2]. Whereas prior studies primarily concentrated on ARFID in children and adolescents [3–5], ARFID can be diagnosed across the lifespan. ARFID is characterized by insufficient energy and/or nutritional intake, leading to significant physical and/or psychosocial impairment, not motivated by body image disturbances or weight-loss intentions, but rather by a lack of interest in eating, sensory characteristics of food, or fear of aversive consequences of eating [2]. While early-onset ARFID was found to be predominately characterized by lack of interest in eating, sensory sensitivity, or combinations [6–8], onset past the age of 18 years has been associated with a higher prevalence of fear of aversive consequences [9]. The overall prevalence of ARFID symptoms in population-based adult samples ranges between 0.3 and 4.8% [10], mostly obtained by self-report. In contrast, in clinical adult samples, ARFID prevalence amounted up to 11% [11], with higher proportions of women with ARFID seeking treatment (e.g., [12]). Among frequently identified comorbidities of adults with ARFID are autism spectrum disorder [12], anxiety disorders [3], obsessive-compulsive disorder (OCD; [9]), and low bone mineral density [13, 14].

Beyond research on comorbid conditions in ARFID, evidence on the specific individual impairment in adults with ARFID remains scarce. A better understanding of ARFID-related impairment in adulthood is relevant because impairment is central to the diagnosis of ARFID yet may not be fully captured by weight status or nutritional compromise alone. While qualitative analyses in children with ARFID reported heightened levels of distress and accommodation impacting daily-life of both the child affected and their family [15, 16], little is known on how ARFID-related impairments shift and manifest in adulthood, when parental accommodation [17] or pressure [18] may vanish, for example, after leaving a parental home. So far, most of the data illustrating the impact of ARFID on adults’ daily life is derived from qualitative case reports. For instance, a 27-year-old woman with ARFID and low appetite since childhood experienced loneliness and social withdrawal after having been teased for her eating behavior [19], while a 41-year-old woman with newly onset ARFID felt too ill to work due to her eating and moved back in with her mother [20]. Similarly, a 25-year-old woman with recently diagnosed ARFID expressed difficulties in her social and work environment [21]. Distress and relationship difficulties were further expressed by a 24-year-old man with longstanding ARFID [22] and a 23-year-old man with ARFID since the age of 16 years described the avoidance of eating-related social encounters [23]. In adults with picky eating and ARFID symptoms (*n* = 13), a qualitative analysis revealed avoidance of social situations due to fear of judgment and stigma [24], while another study (*n* = 9) found that adults with ARFID experienced a “trade-off between safety and freedom,” where food-unknowns limited their travel and social interactions [25]. Overall, these findings exemplify a diverse picture of individual ARFID-related burden involving work, social participation, interpersonal relationships, and everyday autonomy, but they are based primarily on small qualitative studies and case reports.

In addition to psychosocial impairment, ARFID frequently results in physical impairment. For example, adult case reports illustrated nutritional or energy deficiencies causing concentration problems, severe underweight [19, 21, 23, 26] or overweight [22], degeneration of the spinal cord [27], or loss of vision [28]. Consistent with these psychosocial and health impairments, research revealed a reduced (eating-related) quality of life across inpatient and population-based samples of adults with ARFID symptoms compared to healthy controls [9, 29–31].

Unlike research highlighting ARFID’s diagnostic stability (e.g., [7, 32, 33]), little is known on the exact disease progression of ARFID in adults. ARFID commonly develops during early childhood [3, 5, 34, 35] and frequently persists into adulthood – yet ARFID can develop at adult age as well [24, 36]. Preliminary evidence from case studies suggested heterogenous patterns of progression and onset, including a phasic worsening combined with chronic undereating [19], an acute onset after somatic disease (i.e., adenoidectomy, gastroenteritis, pneumonia; [20, 28, 37]) and progressive food restriction since early childhood [27]. Apart from one longitudinal study in adults with ARFID symptoms that identified a positive correlation between emotion regulation difficulties and ARFID symptom severity one year later [38], virtually nothing is known about the factors associated with different ARFID progressions. The identification of such correlates is, however, important for preventive purpose and tailored interventions.

A better understanding of impairment in adults with ARFID is needed because impairment is central to diagnosis, clinical recognition, and treatment planning. In adults, ARFID-related impairment may not always be evident from weight status or medical compromise alone, but may instead manifest in psychosocial functioning, daily routines, social participation, work or education, and quality of life. Clarifying these domains may help distinguish clinically significant ARFID from normative selective eating and identify relevant targets for assessment and intervention. In this context, as the current understanding of ARFID associated impairment and disorder progression of adults with ARFID is based primarily on retrospective chart reviews within specialist eating disorder services [3, 12, 39], single case studies (e.g., [19, 22]), and research on picky eating [31, 40–42], this cross-sectional study aimed to qualitatively explore personal impairment and progression in adults from the community with an interview-based diagnosis of ARFID. Exploratively, this study aimed to identify associations between qualitatively derived groups of progression and sensory sensitivity, temperament, obsessive-compulsive behavior, sociodemographics, and illness duration. Given the heterogeneity of ARFID and the frequent overlap among ARFID presentations [43, 44] it was further explored whether impairment themes, progression groups, and dimensional psychopathology differed between ARFID presentations.

## Methods

### Participants and procedure

Data for this cross-sectional study were collected between November 2023 and February 2025, using a REDCap online survey. Recruitment was conducted via distribution of study flyers, advertisement in online self-help groups (selective eating disorder/ARFID, autism), or the distribution of an online link via social media (e.g., Instagram, Spotify podcast). Initially, all individuals with access to the online questionnaire, age ≥ 18 years old, and who provided informed consent at the start of the questionnaire were eligible to participate in the online questionnaire that took around 45 min to complete. Subsequently, participants were offered a telephone interview to evaluate DSM-5 [2] ARFID diagnostic criteria using the German version of the ARFID Module 2.1 for the Eating Disorder Examination (EDE-ARFID module [45]). Participants’ eligibility for the present study was based on an interview-based diagnosis of ARFID. For compensation purposes, 20€ vouchers were raffled off among online survey participants. Approval for this study was administered by the Ethics Committee of the Medical Faculty of the University of Leipzig, Germany (No. 233/23-ek).

The final sample consisted of *N* = 97 adults with an interview-based diagnosis of ARFID. Of those, *n* = 1 participant did not provide data on impairment and *n* = 5 participants did not provide data on progression, resulting in a total number of *n* = 96 for impairment analysis and *n* = 92 for progression analysis. Recruitment source was assessed during the course of recruitment and was therefore available only for a subset of participants (*n* = 63). Among participants with available information, the most frequently reported recruitment sources were web search (*n* = 23), podcast on ARFID on Spotify (*n* = 11), a national patient organization on ARFID (*n* = 10), other routes (*n* = 9), a private Facebook group for selective eating disorder/ARFID (*n* = 7), and few participants reported Instagram (*n* = 2) or other self-help groups (*n* = 1).

The sample was slightly overrepresented by women (*n* = 68.8%) and had a mean age of 29.5 ± 8.6 years. Regarding ARFID presentations, sensory sensitivity/picky eating was highly prevalent, but there was substantial overlap across clinically relevant presentations. All sample characteristics are listed in Table 1 for the total sample and separately for different ARFID presentations in Table S1 [see Additional file 1].

**Table 1:** Sociodemographic and anthropometric characteristics of adults with ARFID

	*N*	*M* or *n*	*SD* or %
Sociodemographics			
Age, years	97	29.53	8.63
Sex,	96		
Female		66	68.8
Male		28	29.2
Diverse		2	2.1
Nationality	97		
German		90	92.8
Dual		3	3.1
Austrian		3	3.1
Swiss		1	1.0
Family status	96		
Single		78	80.4
Married		14	14.4
Divorced		4	4.1
Education	96		
< 12 School years		36	37.5
≥ 12 School years		60	62.5
Anthropometrics			
BMI, kg/m²	97	24.12	7.71
Underweight (< 18.5)		21	21.6
Normalweight (18.5 – 24.9)		43	44.3
Overweight (25.0 – 29.9)		18	18.6
Obesity (≥ 30.0)		15	15.5
ARFID duration (years)	95	24.50	11.58
Age of onset (years)	97	3.90	0.50
Somatic comorbidities			
Cardiovascular diseases	97	8	8.2
Gastro-intestinal diseases	97	11	11.3
Metabolic diseases	97	15	15.5
Food intolerances/allergies	97	17	17.5
Mental comorbidities			
Depression	97	29	29.9
Neurodevelopmental disorders	97	22	22.7
Anxiety and fear-related disorders	97	23	23.7
EDE-ARFID module core presentation patterns	97		
Only sensory sensitivity		22	22.7
Only lack of interest		0	0.0
Only fear		3	3.1
Sensory sensitivity + lack of interest		23	23.7
Sensory sensitivity + fear		28	28.9
Sensory sensitivity + lack of interest + fear		16	16.5
EDE-ARFID module additional presentation patterns			
No additional presentation		65	67.0
At least one additional clinically relevant presentation		32	33.0
One additional clinically relevant presentation		21	21.6
Two additional clinically relevant presentations		11	11.3
Three additional clinically relevant presentations		0	0.0

Note. Core presentations include sensory sensitivity, lack of interest, and fear of aversive consequences. Additional EDE-ARFID module presentations refer to clinically relevant ratings on emotional problems, ritualistic eating behavior, and/or physical problems presentation scales of the EDE-ARFID module. EDE-ARFID presentation ratings ranged from 0 to 6, with ratings ≥ 4 indicating clinically relevant presentation-related symptoms [45]. Because EDE-ARFID presentations were not mutually exclusive, participants could meet criteria for more than one presentation. *Abbreviation*s: ARFID, avoidant/restrictive food intake disorder; EDE-ARFID module, Eating Disorder Examination ARFID Module; BMI, body mass index (kg/m²)

### Measures

#### ARFID progression and impairment

Participants were asked two open-ended questions as part of the online questionnaire to describe their personal impairment (“What are you currently suffering from most as a result of your avoidant-restrictive eating behavior?”) and the progression of ARFID symptoms over time (“How has your avoidant-restrictive eating behavior been evolved from its initial emergence to the present day?”). All free-text answers were evaluated via qualitative analysis (described below).

#### Avoidant/Restrictive eating behavior

ARFID diagnosis, presentation ratings, and illness duration were assessed using the EDE-ARFID module [45]. The module was administered via phone as a structured clinical interview to evaluate DSM-5 ARFID diagnostic criteria and to confirm the presence of ARFID. Interviews were conducted by trained interviewers who received regular supervision. Diagnostic decisions were discussed within the research team to ensure consistency. In addition, the module includes dimensional ratings of ARFID presentations, including sensory sensitivity, lack of interest, and eating-related fear, and additional presentations emotional problems, ritualistic eating behavior, and physical problems related to eating. Presentation ratings ranged from 0 to 6, with scores of 4 or higher indicating clinically relevant presentation-related symptoms. Because ARFID presentations are not mutually exclusive, presentation ratings were analyzed as overlapping presentation dimensions rather than mutually exclusive subtypes.

The Nine Item ARFID Screener (NIAS; [46]) was employed to assess ARFID symptoms across three subscales containing three items each (NIAS-picky eating, NIAS-appetite, NIAS-fear) on a six-point Likert scale from 0 (strongly disagree) to 5 (fully agree). Higher sum scores per subscale indicate greater symptoms of the respective ARFID presentation. A cut-off score of ≥ 10 for fear of aversive consequences/sensory sensitivity and ≥ 9 for lack of interest indicates a high risk to show respective ARFID presentations [47]. Additional descriptive analyses of NIAS subscale classifications and their convergence with EDE-ARFID module presentation ratings are presented in Table S2 [see Additional file 2]. Internal consistency of the subscales was acceptable to good as indicated by Cronbach’s α and McDonald’s ω for picky eating (α = 0.75; ω = 0.83), appetite (α = 0.86; ω = 0.87), and fear of aversive consequences (α = 0.87; ω = 0.88).

The duration of ARFID symptoms was assessed as part of the diagnostic interview [45] and reported in months.

#### Food neophobia

Reluctance and avoidance towards novel foods was assessed via the 10-item self-report Food Neophobia Scale (FNS; [48]). Each item is answered on a five-point Likert scale from 1 (disagree strongly) to 5 (agree strongly; [49, 50]) with higher total sum scores indicating greater food neophobic symptoms. Internal consistency was good (α = 0.87, ω = 0.88).

#### Obsessive compulsive behavior

Obsessive compulsive personality traits were measured using the 48-item Five Factor Obsessive Compulsive Inventory-Short Form (FFOCI; [51]) comprising 12 subscales consisting of four items each, of which five subscales — Inflexibility, Excessive Worry, Risk Aversion, Perfectionism, Fastidiousness — were utilized for this study. The items are rated on a five-point Likert scale ranging from 1 (strongly disagree) to 5 (strongly agree) with a higher sum score indicating a higher level of the respective obsessive-compulsive characteristic. Internal consistency of the subscales was good for Excessive Worry (α = 0.83; ω = 0.83), acceptable for Risk Aversion (α = 0.76; ω = 0.76), and almost acceptable for Inflexibility (α = 0.68; ω = 0.70), Perfectionism (α = 0.69; ω = 0.64), and Fastidiousness (α = 0.70; ω = 0.69).

#### General sensory sensitivity

The self-report Sensory Summary [52] was utilized to assess sensory sensitivity. It comprises six items to record sensitivity regarding the sensory stimuli of hearing, smell, taste, touch, sight, and texture. The items are rated on an analogue scale ranging from 0 (hyposensitive) to 10 (hypersensitive), with higher total mean scores indicating greater sensory sensitivity. Internal consistency was almost acceptable (α = 0.67, ω = 0.65).

#### Temperament

The two subscales ‘sociability’ and ‘discomfort’ from the Adult Temperament Questionnaire (ATQ; [53]) short form were utilized to assess self-regulatory processes additionally to constitutionally based individual reactivity. The 11 items are rated on a seven-point Likert scale from 1 (not at all applicable) to 7 (completely applicable) with higher mean scores indicating a greater respective characteristic. The internal consistency of the subscales was good for Discomfort (α = 0.80; ω = 0.81) and acceptable for Sociability (α = 0.75; ω = 0.77).

#### Translation and cultural adaption

In accordance with WHO guidelines [54, 55], the items and instructions of the NIAS, FNS, Sensory Summary, and FFOCI were translated into German by the authors and back translated by an independent bilingual English native speaker.

#### Sociodemographics and Anthropometrics

Age, gender (male, female, diverse), height (cm) and weight (kg) to calculate the body mass index (BMI; kg/m2), marital status (single, married, divorced), and highest level of school education (≥ 12 school years, < 12 school years) were assessed via self-report.

### Data analytic plan

#### Qualitative analysis

An inductive methodological combination of thematic and content analysis elements was conducted to assess progression and impairment of adults with ARFID [56, 57], as the inductive approach is deemed to be the most suitable for under-researched areas [58]. The questionnaire responses on impairment and progression were repeatedly read and condensed into meaning units (thematically structured and abbreviated free-text responses) by members of the research team as described below. These meaning units were further condensed into concise word codes and these were grouped into subcategories by identifying similar codes. Finally, the subcategories were synthesized into broader categories or themes, when appropriate [56]. To ensure accuracy and consistency, two different raters (MD candidate, B.Sc. psychology) condensed all open responses to meaning units and word codes, following [59]. Additionally, two more coders (MD candidate, PhD psychologist) analyzed a subset (15%) of open responses from participants, each condensing content to meaning units and further formulating word codes. Discrepancies in meaning units and codes were consecutively discussed by all four coders and aberrated until consensus and data saturation was found. Subcategories, categories, and themes were established within the entire research team and revised until consensus was found. Because participants often described impairment across multiple areas simultaneously, multiple categories and themes could be assigned to a single participant. An example of a coding process is shown in Table S3 [see Additional file 3].

#### Quantitative analysis

To assess differences between progression groups in sociodemographic characteristics (age, gender, nationality, family status, education, BMI, illness duration), pairwise comparisons were conducted. Illness duration was defined as current age minus age of onset. To prevent overestimating illness duration due to older age, illness duration was calculated as the proportion of lifetime lived with ARFID, ranging from 0 to 100, with higher scores reflecting more lifetime lived with ARFID. Further, pairwise comparisons were performed for avoidant/restrictive eating behavior (NIAS subscales, EDE-ARFID module presentations ratings, FNS total score), sensory sensitivities (Sensory Sensitivity mean score), temperament (ATQ subscales: Discomfort, Sociability), and obsessive-compulsive traits (FFOCI subscales: Inflexibility, Excessive Worry, Risk Aversion, Perfectionism, Fastidiousness).

Effect sizes were calculated using Cramér’s *V* for categorical variables and Cohen’s *d* for continuous variables. Following [60] guidelines, Cramér’s *V* and Cohen’s *d* were interpreted as small (0.10, 0.20), medium (0.30, 0.50), and large (0.50, 0.80).

All statistical analyses were conducted using IBM^®^ SPSS Statistics^®^ version 29.0.

## Results

### Qualitative results for impairment

A total of seven themes of personal impairment were identified (see Table 2). Participants most frequently reported impairment by ARFID’s effects on health (*n* = 66, 68.8%), impairment of lifestyle (*n* = 38, 39.6%), and the eating behavior itself (*n* = 33, 34.4%). Less frequently reported impairment themes were social responses (*n* = 23, 24.0%), partnership (*n* = 13, 13.5%), parenthood (*n* = 8, 8.3%), and experiences with practitioners (*n* = 2, 2.1%). Identified themes were divided in categories or subcategories (see Table 2). All themes, categories, and subcategories are listed in Table 2 for the total sample. In addition, themes are separately listed for different ARFID presentations in Table 3. These descriptive analyses indicated that impairment theme frequencies were relatively balanced across ARFID presentations.

**Table 2:** Themes, categories, and subcategories of impairment in adults with ARFID

Topic	Sub(categories)	Quote		Frequency, *n* (%)*
**Health-Related Impairment**	The theme “health-related impairment” reflects participants’ reports of physical and psychological consequences associated with ARFID. Physical consequences included micronutrient deficiencies, weight-related problems (underweight, overweight, and impeded weight change), autonomic symptoms, and reduced capacity for physical activity. Participants also described burden related to medication. Psychological consequences encompassed changes in affect, social-emotional insecurity, reduced quality of life, shame about eating behaviors, social withdrawal, and maladaptive coping.			66 (68.8)
	***Physical Consequences***			44 (45.8)
	**Weight Problems.** “Weight problems” describes a change in body weight (underweight, overweight) because of eating behavior and insufficient skills to change body weight.	*…High weight gain due to high-carbohydrate safe foods and eating out of frustration …*		21 (21.9)
		*…It is very frustrating for me not to be able to gain weight…*		
	**Autonomic Changes.** “Autonomic changes” summarizes the effects of eating behavior on vegetative bodily functions such as physical energy levels or appetite.	*…I often feel as if my body is running on fumes…*		16 (16.7)
		*…fatigue and listlessness…*		
		*…energy and insomnia…*		
	**Difficult Micronutrient Supply.** “Difficult micronutrient supply” describes the impairment of those affected by a reduced nutrient intake and the resulting nutrient deficiencies.	*…I suffer from nutrient deficiency*,* for instance*,* I experience an iron deficiency very regularly (almost chronic) since 7 years. This is regularly counteracted with iron medication. Vitamin D and folic acid deficiency were also diagnosed this year…*		5 (5.2)
	**Difficulties With Sports.** “Problems with sports” includes the impairment of not being able to gain muscle mass and to perform at full capacity during sports due to the eating behavior.	*…What impairs me most is a reduced sports performance*,* I would like to do more sports*,* build up muscles etc.*,* but unfortunately*,* I find this very difficult…*		5 (5.2)
	**Skin Complaints.** “Skin complaints”, as distinct from the category “secondary diseases”, covers nutrition-related effects on the skin of those affected.	*…I would like to get my acne under control with the right diet…*		3 (3.1)
		*…The effects of my eating behavior on my skin (uneven and pale)…*		
	**Secondary Diseases.** “Secondary diseases” includes general, physical secondary conditions that result from selective eating behavior.	*…in the meantime*,* I get physical problems (chronic pancreatitis*,* fatty liver…)*		3 (3.1)
	**Metabolic Consequences.** “Metabolic consequences” describes the impairment by diet-related metabolic changes.	*…Experiences of high blood pressure as a consequence to my eating behavior …*		2 (2.1)
		*… higher cholesterol levels…*		
	**Complicated Medication.** “Complicated medication” describes difficulties in taking medication orally and the associated health consequences as well as the lack of compliance.	*…swallowing pills is not an option either…*		2 (2.1)
	**Dental Consequences.** “Dental consequences” covers the impairment caused by dental problems resulting from the eating behavior.	*…I suffer from the condition of my teeth (I have been going to the dentist approx. every 3–4 months since 2021 due to severe demineralization of my entire teeth)…*		2 (2.1)
	**Weakened Immune System.** “Weakened immune system” describes the negative effects of the eating behavior on immune function.	*…Physical secondary diseases impair me. I have always had an extremely poor immune system and accordingly took antibiotics 3–5 times a year in my childhood and youth…*		2 (2.1)
		*…I`m often ill…*		
	***Psychological Consequences***			42 (43.8)
	**Affective Consequences.** “Affective consequences” summarizes the effects of the eating behavior on the mental state and mood.	*…I often experience a slight depressive mood when everyone can eat something but me…*		13 (13.5)
		*…I can’t do anything “good” for myself because of this and THAT makes me very sad…*		
	**Inadequate Coping Strategies.** “Inadequate coping strategies” describes participants’ perceived helplessness in attempting to change their eating behavior.	*…I would like to eat more varied and healthier food*,* but it’s just not possible from a sensory point of view…*		9 (9.4)
		*…A feeling of helplessness*,* of not being able to influence or change anything…*		
	**Social-Emotional Insecurity.** “Social-emotional insecurity” describes participants’ insecurity to stand out negatively in social situations and the associated psychosocial stress experienced. This results in uncertainty among those affected about how they are perceived in social interaction.	*…I fear embarrassing myself in public (e.g. having to go to the toilet at an inappropriate time and not being able to eat when you spontaneously go to a restaurant)…*		7 (7.3)
		*…For example*,* I put pressure on myself before eating out with others because I’m afraid I won’t find anything. For me*,* this is also a reason to look for excuses from the meeting as such…*		
		*…I suffer when others “force” food on me to try. I find it difficult to say “no” to this*,* which is why I often make excuses in such situations so that I don’t have to eat what has been forced upon me…*		
	**Shame.** “Shame” describes the feeling of shame experienced by participants in social situations or because they are unable to eat in ways that meet social expectations.	*…I feel ashamed to order something or prepare something to eat…*		7 (7.3)
		*…I need an extra dish everywhere. I’m ashamed…*		
		*…I’m embarrassed that as an adult academic I only eat junk food*,* as if I had a nutritional deficiency or as if I were a 6- year-old child who was allowed to go shopping alone…*		
	**Self-Devaluation.** “Self-devaluation” describes participants' personal devaluation because of their eating behavior and the inability to change it.	*…I hate myself for not being able to eat “normally”. I feel like an alien and devalue myself…*		5 (5.2)
		*…the impression that something is wrong with me impairs me…*		
	**Food-Related Fears.** “Food-related fears” describes participants’ impairment due to fears of the food itself, fear of subsequent physical reactions, and fear of negative experiences when eating with others.	*…The fear I have when I have to eat with other people and I don’t know what’s on the menu*,* or I know I don’t like the food…*		4 (4.2)
		*…I would say under the fear that the food is bad or mouldy*,* for example…*		
		*…I put a lot of pressure on myself before going out to eat with others because I’m afraid I won’t find anything…*		
	**Social Isolation.** “Social isolation” describes participants’ feelings of not being socially accepted and integrated as a consequence of their eating behavior.	*…There are hardly any social contacts left…*		3 (3.1)
		*…As a result of my eating behavior*,* I have been socially excluded from my peers since early childhood and it makes me very lonely…*		
	**Concern About Health Consequences.** “Concern about health consequences” describes the concern of participants that their eating behavior will lead to future health consequences.	*…I worry that my weight loss will eventually become critical…*		3 (3.1)
		*…Concern about health consequences…*		
	**Reduced Quality of Life.** “Reduced quality of life” refers to participants’ decreased capacity to enjoy life and engage in it fully.	*…I can’t enjoy my life…*		2 (2.1)
		*…I have to live life differently*,* than people around me…*		
	**Psychological Consequences (Unspecified).**	*…psychological consequences…*		1 (1.0)
**Impairment of Lifestyle**	The theme “lifestyle impairment” captures restrictions in occupational, social, and leisure-related daily life, as well as mobility-related limitations (e.g., during travel) and financial or organizational challenges associated with eating behaviors. In contrast to “social isolation,” which reflects the subjective experience of feeling socially isolated, this category refers to concrete restrictions in participation in everyday social activities.			38 (39.6)
	***Everyday Restrictions***			38 (39.6)
	**… Social.**	*…I restrict myself and those around me in many activities…*		27 (28.1)
		*…I avoid social gatherings (e.g.*,* meetings with colleagues) where shared meals are involved…*		
		*…I can’t go out for dinner with friends…*		
	**…Professional.**	*…I suffer from social restrictions. For instance*,* I never really got on with the team at work*,* as I avoid almost all meetings outside because of food…*		15 (15.6)
		*…I choose a different job than firefighter due to eating together at the station…*		
		*…At work*,* I’d like to eat there*,* but I’m afraid to…*		
	**…Vocational/Travel.**	*…the constant planning to see if I can find something to eat outside. This is very difficult*,* especially on vacation. Traveling to many countries is therefore hardly possible…*		9 (9.4)
		*…I have difficulties on vacation. I would like to get to know other cultures better*,* travel more*,* but usually the only safe food is bread and chips…*		
	**…Financially/Organizational.**	*…It’s annoying to always stress yourself out when you go out to eat*,* first analyzing the whole menu online before you go and then realizing that they don’t even have fries or at least any side dish you like…*		4 (4.2)
	**…Leisure Time.**	*…Almost every week there are situations in which I am restricted by my eating behavior (leisure activities)…*		3 (3.1)
		*…restrictions on…leisure activities (it is impossible being outdoors longer than 6 h)…*		
**Eating Behavior**	The theme “eating behavior” describes the impairment of participants as a consequence to eating too little, unhealthy, or monotonous food. The loss of pleasure is also a key issue.			33 (34.4)
	**Restriction of Food Variety.** “Restriction of food variety” summarizes the impairments caused by a very limited selection of acceptable foods. Specifically, participants suffer from the monotony and one-sidedness of the food, combined with the unfulfilled desire to be able to eat more food.	*…I also struggle with myself because I would like to eat more varied food (I have the impression that my life would be easier then…)*		14 (14.6)
		*…low variance in food intake (number of different dishes)…*		
	**Unhealthy Diet.** “Unhealthy eating behavior” describes how participants felt impaired by the fact of being aware to not be able to eat a balanced and healthy diet. This includes the desire to eat more healthily.	*…I would like to be able to eat healthy food without having to suppress a nausea at the thought of it…*		12 (12.5)
		*…I am very overweight and would like to lose weight. But how can I do that if I CAN only eat unhealthy things? Changing my diet is simply not possible…*		
	**Restriction of Food Amount.** “Restriction of food quantity” describes the impairment of only being able to eat a little and the fears of eating too little.	*…I often can’t eat enough as a result and then have little energy…*		10 (10.4)
		*…I can’t finish the whole portion…*		
		*…I currently often suffer from hunger (and can eat nothing or almost nothing)…*		
	**Loss of Pleasure.** “Loss of pleasure” describes the impairment caused by a loss of enjoyment and pleasure in eating.	*…I experience monotony and see no pleasure in eating…*		5 (5.2)
		*…Many dishes look delicious and because of the restrictions I have the feeling that I’m missing out on something…*		
**Social Responses**	The theme “social responses” captures the distress participants experience in response to how people outside the immediate family or intimate relationship react to their eating behavior. Central aspects include criticism, lack of understanding, and pressure to explain or justify their eating behavior.			23 (24.0)
	**Pressure to Justify.** “Pressure to justify” describes participants’ experiences of pressure to justify their eating behavior to others.	*…Having to justify myself to others and having to listen to people telling me that I’m making a mistake…*		8 (8.3)
		*…I suffer most from having to constantly explain myself at the table with “unknown” people…*		
	**Lack of Understanding From Others.** “Lack of understanding from others” describes the experience of being misunderstood or ignored by others. This can manifest itself in conflicts with others, for example.	*…Food is a very central topic for most people*,* and they often show little understanding for my eating behavior*,* even if I explain it or hide it…*		7 (7.3)
		*…I can’t cope with the opinions of others who don’t understand that I don’t eat like this on purpose…*		
		*…In my environment*,* in the family*,* it is not accepted or acted as if it were new information…*		
	**Criticism.** “Criticism” describes the critical and sometimes reproachful way in which others deal with participants’ eating behavior.	*…It was great*,* for example*,* to hear again at Christmas*,* what do we want to eat? WE eat everything*,* YOU don’t!…*		4 (4.2)
		*…I can’t go out to dinner with friends without having to endure a lot of questions and condemnations…*		
	**Social Stigmatization.** “Social stigmatization” describes the experience of social stigmatization experienced by participants, for example, due to the prejudices of others.	*…I feel socially ostracized…*		3 (3.1)
		*…It’s extremely annoying when you get to know someone new and then have to explain your eating behavior to them*,* even though they don’t believe you anyway and just think you’re picky…*		
		*…I suffer from social stigmatization…*		
	**External Pressure.** “External pressure” describes the experience of being forced to eat by others and a general feeling of pressure.	*…my family and my relationship person are worried - regarding my family I always think to myself* “*I’m doing my best anyway - eating more is easier said than done"…*		2 (2.1)
		*…The social pressure on me…*		
**Partnership**	The theme “partnership” covers how the eating behavior restricts activities within the partnership (e.g., cooking together) or leads to conflicts. Concerns about the future viability of a partnership are also included within this theme.			13 (13.5)
	**Burden on Partnership.** “Burden on partnership” describes how participants feel burdened and affected by food-related discussions, conflicts, and disagreements within the partnership.	*…My eating behavior has a negative impact on my relationship…*		8 (8.3)
		*…my husband*,* who loves to experiment*,* has to cut back a lot…*		
		*…I also suffer from how difficult the food discussion sometimes is between me and my boyfriend…*		
	**Restriction of Activity in Partnership.** In contrast to “burden on partnership”, “restriction of activity in partnership” describes the fact that people with ARFID feel restricted in partnership activities such as eating out and cooking together.	*…The desire to cook with a partner…*		4 (4.2)
		*…I can never really go out to eat with my girlfriend*,* it’s a burden for both of us…*		
	**Future Partnership.** “Future partnership” describes the concern about being able to enter a long-term relationship while having this eating behavior.	*…I am unsure about my eating behavior in relation to a longterm relationship…*		1 (1.0)
**Parenthood**	The theme “parenthood” includes the subcategories of starting a family and parental role model function. This addresses both participants’ concern about not being a good role model for a child or being prevented from starting a family in the future due to eating behavior, e.g., by endangering a pregnancy.			8 (8.3)
	**Parental Role Model Function.** “Parental role model function” describes the experienced impairment of participants in their role as parents. It results from not being able to be a good role model for a healthy, balanced diet due to their eating behavior.	*…Most of all I worry about unintentionally teaching my daughter this eating behavior because I can’t set an example for her to eat healthily…*		6 (6.3)
		*…I’m afraid of being a bad role model for my children…*		
	**Starting a Family.** “Starting a family” deals with the question of how participants are able to perceive or raise a child in the future. The impairment results from the fact that the ability to have or raise children in the future is perceived as impaired.	*…I couldn’t imagine raising children with an eating disorder…*		3 (3.1)
		*…Knowing that my eating behavior could possibly endanger a pregnancy…*		
**Experiences With Practitioners**	The theme “experiences with practitioners” describes how participants feel affected by ignorance and judgment by medical professionals.			2 (2.1)
	**Medical Handling of Eating Behavior.** The category “medical handling of eating behavior” describes participants’ experiences of ignorance and judgment by doctors.	*… The ignorance and lack of knowledge in society and among doctors about this topic impairs me most…*		2 (2.1)

**Table 3:** Impairment characteristics among ARFID presentations

	Total sample	NIAS			EDE-ARFID module					
		Picky eating (*n* = 88)	Appetite (*n* = 30)	Fear (*n* = 11)	Sensory sensitivity (*n* = 93)	Eating-related anxiety (*n* = 47)	Lack of interest (*n* = 38)	Emotional problems (*n* = 17)	Ritualized eating behavior (*n* = 13)	Physical problems (*n* = 13)
	*n* (%)	*n* (%)	*n* (%)	*n* (%)	*n* (%)	*n* (%)	*n* (%)	*n* (%)	*n* (%)	*n* (%)
Health impairment	66 (68.8)	59 (67.0)	23 (76.7)	10 (90.9)	64 (68.8)	33 (70.2)	28 (73.7)	10 (58.8)	10 (76.9)	11 (84.6)
Impairment of lifestyle	38 (39.6)	36 (40.9)	8 (26.7)	4 (36.4)	37 (39.8)	19 (40.4)	11 (28.9)	8 (47.1)	3 (23.1)	3 (23.1)
Eating bahavior	33 (34.4)	29 (33.0)	11 (36.7)	6 (54.5)	31 (33.3)	16 (34.0)	12 (31.6)	2 (11.8)	3 (23.1)	5 (38.5)
Social responses	23 (24.0)	22(25.0)	5 (16.7)	2 (18.3)	23 (24.7)	10 (21.3)	9 (23.7)	8 (47.1)	3 (23.1)	1 (7.7)
Partnership	13 (13.5)	12 (13.6)	3 (10.0)	1 (9.1)	13 (14.0)	6 (12.8)	4 (10.5)	1 (5.9)	1 (7.7)	1 (7.7)
Parenthood	8 (8.3)	8 (9.1)	3 (10.0)	0 (0.0)	8 (8.6)	3 (6.4)	3 (7.9)	1 (5.9)	2 (15.4)	0 (0.0)
Experiences with practitioners	2 (2.1)	2 (2.3)	1 (3.3)	0 (0.0)	2 (2.2)	1 (2.1)	0 (0.0)	1 (5.9)	0 (0.0)	0 (0.0)

Note. The percentages reflect the proportion of individuals scoring both above the cut-off for the respective ARFID presentation and respective impairment theme, compared to all individuals scoring above the cut-off for a respective ARFID presentation. In line with recommendations by Burton Murray et al. [47], clinically relevant symptomology was defined using NIAS subscale cut-offs of ≥ 10 for picky eating and fear, and ≥ 9 for appetite. Clinically relevant EDE-ARFID module presentations symptomology was indicated by scores ≥ 4 [45]. *Abbreviations*: ARFID avoidant/restrictive food intake disorder; EDE, Eating Disorder Examination; NIAS, Nine-Item ARFID Screener

#### Health-related impairment

Regarding health-related impairment, participants were affected by both physical (*n* = 44, 45.8%) and mental health consequences (*n* = 42, 43.8%).

##### **Physical consequences**

Among physical consequences, the majority of participants reported weight-related problems (*n* = 21, 21.9%), including overweight and underweight-related impairment, or experienced difficulties of maintaining a stable weight. In addition, *n* = 16 (16.7%) reported changes in vegetative functions (e.g., loss of appetite, insomnia) to be most impairing. Less frequently reported (*n* ≤ 5) were challenges with physical exercise, impeded micronutrient supply, changes in skin appearance, difficulties with medication intake, dental problems, weakened immune system, and metabolic consequences, such as hypertonia, or secondary diseases (steatosis hepatis and pancreatitis).

Given that weight-related impairment was reported most frequently, this participant offered insight into how this impairment manifests:

*“… I am very overweight and would like to lose weight. But how can I do that if I CAN only eat unhealthy things? Changing my diet is simply not possible…” (woman*,* 20 years)*.

##### **Psychological consequences**

Participants were most likely impaired by affective consequences, specifically, reduced mood and mental capability (*n* = 13, 13.5%), inadequate coping strategies (*n* = 9, 9.4%), social-emotional insecurities (*n* = 7, 7.3%), such as feeling insecure of being negatively perceived by others and eating behavior-related shame (*n* = 7, 7.3%). Less frequently reported categories of psychological impairment (*n* ≤ 5) were self-devaluation, food-related fears, social isolation, concerns about health consequences, reduced quality of life, and psychological consequences that were not further specified.

Concerning shame, this participant articulated it in the following manner:

*“… I’m embarrassed that as an adult academic I only eat junk food*,* as if I had a lack of knowledge about nutrition or as if I were a 6-year-old child who was allowed to go shopping alone …” (women*,* 33 years)*.

#### Eating behavior

Regarding eating behavior, participants indicated impairment associated with limited food variety (*n* = 14, 14.6%), an unhealthy diet (*n* = 12, 12.5%), reduced food amount (*n* = 10, 10.4%), and loss of pleasure (*n* = 5, 5.2%) characterized by the fear of missing out on culinary experiences and monotony of their own food selection, as portrayed by this participant:

*“…I also struggle with myself because I would like to eat more varied food. I have the impression that my life would be easier then…” (woman*,* 30 years)*.

#### Impairment of lifestyle

On the subject of lifestyle impairment, social (*n* = 27, 28.1%), professional (*n* = 15, 15.6%), vocational/travel (*n* = 9, 9.4%), financial/organizational (*n* = 4, 4.2%), and leisure time (*n* = 3, 3.1%) everyday restrictions were reported. In the majority of cases, social impairment was characterized by difficulties in dining out and an impaired capacity to engage in shared activities, as this participant explained:

*“… No trips with friends. No long-distance travel. Other job choice than firefighter due to eating together at the station …” (man*,* 28 years)*.

Or as another participant described:

*“… social restrictions (work*,* friends); it’s annoying to always be restricted as soon as I want/need to eat outside the home …” (woman*,* 31 years)*.

#### Social responses

Among participants reporting impairment associated with responses of the social environment to the eating behavior, most were pressured by justifying their eating behavior (*n* = 8, 8.3%) and a lack of understanding from others (*n* = 7, 7.3%) including misunderstanding eating difficulties as pickiness or reacting with ignorance. Less frequently reported (*n* < 5) impairment was related to criticism, social stigmatization, and external pressure to eat, as this participant portrayed:

*“… It’s also extremely annoying when you get to know someone new and then have to explain it to them*,* even though they don’t believe you anyway and just think you’re picky…” (man*,* 18 years)*.

#### Partnership

Concerning partnership, participants reported impairment including burden on partnership (*n* = 8, 8.3%), such as discussions, conflicts, and disagreements over food selection, the restriction of activities in partnership (*n* = 4, 4.2%), for example, shared cooking or dining out, and concerns about future partnership due to ARFID (*n* = 1, 1.0%). This participant exemplified the impact of his eating behavior on his partnership:

*“…When it comes to my partner*,* I feel like a burden — as if I only create extra work and worry. For example*,* my circulatory problems when we’re out*,* my lack of energy*,* or the fact that I can’t cook and usually only manage pasta with tomato sauce…” (man*,* 22 years)*.

#### Parenthood

Concerning parenthood, participants reported impairment regarding their parental role model function (*n* = 6, 6.3%) and the ability to start a family (*n* = 3, 3.1%) including worries to jeopardize a pregnancy and inability to raise future kids. This mother shared her experiences of how ARFID influences her parenting:

*“…What worries me most is unintentionally passing on this eating behavior to my daughter*,* since I can’t exemplify her healthy eating. So far*,* she eats a balanced diet (she especially loves fruit)*,* but is now entering the typical picky eating phase*,* and I’m afraid it might stick. As a single parent*,* there’s also no one else sitting at the table with her…” (woman*,* 36 years)*.

#### Experiences with practitioners

A minority of participants (*n* = 2, 2.1%) reported impairment regarding the medical handling of restrictive eating behavior as they experienced judgement and ignorance of practitioners, as this participant described:

*“…The stigmatization of my daughter by doctors and therapeutics because she displays similar eating behavior as myself*,* a therapy based on the principle ‘if the child isn’t offered any food*,* sooner or later it will eventually start to eat’”. This existential withdraw of food was already the most accepted practice during my own childhood and left me traumatized…” (woman*,* 55 years)*.

### Qualitative results for ARFID progression

A total of four progression categories were identified including improved (*n* = 38, 41.3%), deteriorated (*n* = 17, 18.5%), consistent (*n* = 30, 32.6%), and phasic progressions (*n* = 7, 7.6%). Due to small group size, the ‘phasic’ group was not included in the main comparative group analyses but is portrayed in the supplement, see Table S4 [see Additional file 4] and S5 [see Additional file 5].

#### Improved

If improvement of variety and/or quantity of food intake was mentioned in the absence of worsening of other symptoms, this was evaluated as an overall improvement. Instances in which the free text response exclusively described an improvement in symptoms, such as a decrease in nausea when confronted with unacceptable foods or an improvement in trying new foods, were also rated as an overall improvement. This participant gives an example of an improved progression:

*“…My diet as a child was more restrictive compared to now. Meanwhile I try more unknown foods*,* but I’m not always successful. However*,* the range of accepted foods has widened…” (woman*,* 29 years)*.

#### Deteriorated

A decrease in food variety and/or quantity was evaluated as a deterioration of ARFID symptoms. Concordant with the assessment of progression improvement, responses that exclusively provided information regarding a deterioration of symptoms, such as increased sensory sensitivity to food or weight changes as a result of an imbalanced, high-calorie nutrition, were rated as deterioration. This participant gives an example of a deteriorated progression:

*“…It’s getting worse and worse. I often can’t even eat until I’m full because I’m so sick of the foods I can eat…” (man*,* 30 years)*.

#### Consistent

A consistent progression was evaluated if both consumed food variety/quantity and symptoms exhibited were consistent. In the event of consistent symptoms, yet a decrease or increase in variety or quantity was reported, the symptom was categorized as an overall improvement or deterioration as described above. This participant gives an example of a consistent progression:

*“…Actually*,* for me there is no real change. I know what I can or like to eat as soon as I see the food. There are no better or worse days. Over time*,* of course*,* I’ve gotten to know more different foods. I would really like to eat most of these foods*,* but sadly it just doesn’t work for me that way…” (woman*,* 31 years)*.

#### Phasic

Alternating phases of severe and reduced restriction in terms of both food quantity and variety were evaluated as phasic progression, resulting in categories of phasic progression with and without remission. This participant gives an example of a phasic progression:

*“…It’s a constant up and down. There are phases of me courageously trying new foods. When I experience discomfort from a certain food and this happens frequently*,* I go back to eating only safe foods for a while. This pattern is also true for phases of mental crisis. In times of mental crisis*,* I reduce the amount and selection of foods extensively – it gets more compulsive…” (woman*,* 51 years)*.

### Clinical characteristics of different ARFID progressions

Regarding sociodemographic differences between progression groups, both the improved and the consistent group exhibited longer illness duration and later onset of ARFID than individuals within the deteriorated group (large effects), see Table 4. Participants in the improved group were older (medium effect) and reported a higher level of education compared to those in the deteriorated group (medium effect). Those in the deteriorated group had lower levels of education than those of the consistent group (medium effect). No other at least medium-sized effects were seen.

**Table 4:** Comparisons of progression groups (improved, deteriorated, and consistent) in sociodemographic variables

	Improved (*n =* 38)	Deteriorated (*n* = 17)	Consistent (*n* = 30)	Improved vs. Deteriorated	Improved vs. Consistent	Deteriorated vs. Consistent
	M (SD) or *n* (%)	M (SD) or *n* (%)	M (SD) or *n* (%)	Effect sizes (d/V) [95% CI]		
Sociodemographics						
Age, years	31.63 (9.63)	26.88 (4.23)	27.83 (8.17)	0.56 [-0.02, 1.13]	0.42 [-0.06, 0.89]	-0.13 [-0.72, 0.45]
Gender^1^				0.06 [0.01, 0.34]	0.15 [0.01, 0.40]	0.08 [0.01, 0.38]
Female	29 (76.3)	12 (70.6)	17 (58.6)			
Nationality^2^				0.09 [0.07, 0.19]	0.10 [0.01, 0.30]	0.16 [0.09, 0.27]
German	36 (94.7)	16 (94.1)	28 (93.3)			
Education level^3^				0.45 [0.18, 0.69]	0.11 [0.01, 0.34]	0.36 [0.08, 0.62]
≥ 12 school years	28 (73.7)	5 (29.4)	20 (66.7)			
Marital status^4^				0.09 [0.01, 0.31]	0.11 [0.01, 0.33]	0.01 [0.01, 0.33]
Single	30 (78.9)	15 (88.2)	24 (82.8)			
Married	7 (18.4)	2 (11.8)	3 (10.3)			
Anthropometrics						
BMI, kg/m²	23.70 (5.01)	25.04 (9.24)	24.04 (10.02)	-0.20 [-0.77, 0.43]	-0.09 [-0.56, 0.38]	0.06 [-0.52, 0.65]
ARFID duration (% of lifetime)	91.31 (13.24)	74.55 (21.17)	91.6 (10.6)	1.03 [0.43, 1.62]	-0.02[-0.50, 0.45]	-1.20 [-1.72, -0.48]
Age of onset (years)	2.47 (2.84)	6.65 (5.38)	2.40 (3.87)	-1.09 [-1.68, -0.48]	0.02 [-0.45, 0.50]	0.93 [0.31, 1.55]

Note. Cohen’s *d* and Cramér’s *V* were used to assess effect sizes in group comparisons, interpreted as small (*d* = 0.2; *V* = 0.1), medium (*d* = 0.5; *V* = 0.3) and large (*d* = 0.8; *V* = 0.5). 95% percentile confidence intervals for Cramer’s *V* were estimated via bootstrapping (5,000 resamples). *Abbreviations*: ARFID, avoidant/restrictive food intake disorder; BMI, body mass index (kg/m²)

^1^ Gender was dichotomized into *female* and *male* for analysis

^2^ Nationality was grouped into *German* and *Other* due to low frequencies in individual categories

^3^ Education level was dichotomized in *≥ 12 years of school* and *< 12 years of school*

Regarding avoidant/restrictive eating behaviors, the improved group scored lower compared to the deteriorated group on the NIAS Fear subscale (medium effect; Table 5). Moreover, the improved and consistent group scored lower on eating-related anxiety (EDE-ARFID module, medium effect; Table 5) and the consistent group scored higher on ritualized eating behavior (EDE-ARFID module) compared to the deteriorated group (medium effect; Table 5). No other group differences with at least medium effect were found.

**Table 5:** Comparisons of progression groups (improved, deteriorated, and consistent) in clinical variables

	Improved (*n =* 38)	Deteriorated (*n* = 17)	Consistent (*n* = 29)	Improved vs. Deteriorated	Improved vs. Consistent	Deteriorated vs. Consistent
	M (SD) or *n* (%)	M (SD) or *n* (%)	M (SD) or *n* (%)	Effect sizes (d/V) [95% CI]		
Avoidant/restrictive behavior						
NIAS picky eating, 0–15	13.74 (1.81)	13.65 (2.10)	13.07 (2.89)	0.05 [-0,52, 0.62]	0.28 [-0.19, 0.76]	0.22 [-0.38, 0.81]
Above cut-off ≥ 10	36 (94.70)	16 (94.10)	27 (90.00)	0.01 [0.01, 0.30]	0.09 [0.01, 0.31]	0.06 [0.01, 0.30]
NIAS appetite, 0–15	5.11 (4.42)	6.31 (4.39)	6.50 (5.70)	-0.27 [-0.85, 0.31]	-0.28 [-0.75, 0.20]	-0.04 [-0.63, 0.56]
Above cut-off ≥ 9	10 (26.3)	4 (25.0)	13 (43.3)	0.01 [0.00, 0.30]	0.18 [0.01, 0.41]	0.18 [0.01, 0.44]
NIAS fear, 0–15	1.97 (3.27)	4.53 (4.40)	3.40 (4.75)	-0.69 [-1.27, -0.11]	-0.35 [-0.83, 0.13]	0.24 [-0.35, 0.83]
Above cut-off ≥ 10	2 (5.30)	2 (11.8)	4 (13.3)	0.12 [0.01, 0.39]	0.14 [0.01, 0.36]	0.01 [0.01, 0.32]
EDE-ARFID module presentations ≥ 4						
Sensory sensitivity	4.47 (0.51)	4.47 (0.80)	4.53 (0.78)	0.01 [-0.56, 0.57]	-0.09 [-0.57, 0.38]	-0.08 [-0.66, 0.51]
Eating-related anxiety	2.29 (1.86)	3.59 (1.58)	2.47 (1.93)	-0.72 [-1.30, -0.14]	-0.09 [-0.57, 0.38]	0.61 [0.01, 1.20]
Lack of interest	2.34 (1.91)	2.71 (1.76)	2.57 (2.14)	-0.19 [-0.76, 0.37]	-0.11 [-0.58, 0.36]	0.07 [-0.52, 0.65]
Emotional problems	1.61 (1.78)	1.35 (1.54)	1.33 (1.45)	0.15 [-0.43, 0.72]	0.16 [-0.31, 0.64]	0.01 [-0.57, 0.60]
Ritualized eating behavior	1.47 (1.50)	0.94 (1.09)	1.83 (1.68)	0.38 [-0.20, 0.96]	-0.22 [-0.70, 0.25]	-0.59 [-1.18, 0.01]
Physical problems	0.95 (1.52)	0.94 (1.44)	1.27 (1.72)	0.00 [-0.56, 0.57]	-0.20 [-0.67, 0.28]	-0.20 [-0.80, 0.40]
Food neophobia, 10–50	42.03 (6.31)	42.76 (4.34)	41.60 (7.89)	-0.13 [-0.69, 0.44]	0.06[-0.41, 0.53]	0.17 [-0.42, 0.75]
Sensory sensitivities						
Sensory summary, 0–10	6.30 (1.35)	5.81 (2.12)	6.25 (1.67)	-0.30 [-0.27, 0.86]	0.03 [-0.44, 0.51]	-0.23 [-0.82, 0.36]
Temperament (ATQ), mean scores						
Discomfort 1–6	4.23 (1.59)	3.82 (1.42)	4.37 (1.20)	0.26 [-0.31, 0.83]	-0.09 [-0.57, 0.38]	-0.42 [-1.00, 0.18]
Sociability 1–5	3.88 (1.34)	3.65 (1.23)	3.67 (1.39)	0.18 [-0.39, 0.74]	0.15 [-0.32, 0.63]	-0.02 [-0.60, 0.57]
Obsessive compulsive behavior (FFOCI), 4–20						
Inflexibility	13.61 (2.83)	12.53 (3.59)	14.07 (3.12)	0.34 [-0.23, 0.91]	-0.15 [-0.63, 0.32]	-0.46 [-1.05, 0.14]
Excessive worry	14.95 (3.62)	16.12 (3.59)	14.90 (4.21)	-0.32 [-0.89, 0.25]	0.01 [-0.46, 0.49]	0.30 [-0.29, 0.89]
Risk aversion	14.58 (3.12)	14.29 (3.95)	14.97 (3.35)	0.08 [-0.48, 0.65]	-0.12 [-0.59, 0.37]	-0.19 [-0.77, 0.40]
Perfectionism	14.08 (3.05)	13.71 (2.93)	14.40 (3.78)	0.12 [-0.44, 0.69]	-0.09 [-0.57, 0.38]	-0.20 [-0.78, 0.39]
Fastidiousness	13.08 (3.22)	12.44 (3.61)	13.97 (4.38)	0.19 [-0.39, 0.77]	-0.23 [-0.71, 0.24]	-0.36 [-0.96, 0.24]

Note. Cohen’s *d* and Cramér’s *V* were used to assess effect sizes in group comparisons, interpreted as small (*d* = 0.2; *V* = 0.1), medium (*d* = 0.5; *V* = 0.3), and large (*d* = 0.8; *V* = 0.5). 95% percentile confidence intervals for Cramer’s *V* were estimated via bootstrapping (5,000 resamples). Cohen’s *d was calculated with* Hedges' correction due to different group sizes. *Abbreviations*: NIAS, Nine-Item ARFID Screener; EDE-ARFID module, Eating Disorder Examination ARFID Module; ATQ, Adult Temperament Questionnaire; FFOCI, Five-Factor Obsessive-Compulsive Inventory

Comparisons of progression groups on ATQ subscales (Discomfort and Sociability) and FFOCI subscales (Inflexibility, Excessive Worry, Risk Aversion, Perfectionism, Fastidiousness) revealed only less than small effects.

## Discussion

Despite increasing scientific recognition of ARFID, most evidence stems from pediatric samples, leaving adult presentations insufficiently characterized including experienced impairment and disorder progression. Uniquely, this mixed-method study in *N* = 97 adults with an interview-based diagnosis of ARFID from the community focused on gaining a nuanced understanding of individual impairment, and progression patterns beyond diagnostic criteria. Based on a mixed thematic-content analysis approach, seven themes of major individual impairment emerged, including (1) health-related impairment concerning physical and mental consequences, (2) impairment of lifestyle including social, professional, vocational/travel, financially/organizationally, and leisure time dimensions, (3) impairment by the eating behavior itself, (4), impairment due to responses of the social environment, (5) partnership, (6) parenthood, and (7) impairment due to experiences with practitioners. Further, four distinct ARFID progression patterns were identified: improved (41.3%), deteriorated (18.5%), consistent (32.6%), and phasic (7.6%) symptomatology. Overall, these findings highlight the heterogeneity of impairment and suggest that ARFID presentation, age, and education level may be associated with progression patterns.

Consistent with meta-analytical evidence and systematic reviews including mostly pediatric and adolescent samples with ARFID [3, 13, 61], physical health problems were highly prevalent in this sample and experienced by 45.8% as the main source of distress. While underweight – a diagnostic criterion of ARFID often met in treatment seeking samples – was reported by 5.9% as impairing, weight problems as a result of being overweight emerged as a predominant concern in 16.7% of participants. As 34.1% of the sample exhibited a BMI > 25 and 21.6% exhibited a BMI < 18.5 kg/m² in this nonclinical sample, the proportion of those perceiving their weight status as impairing was notably higher in those presenting as being overweight than in those presenting as being underweight. Especially in adults, recent studies recognized being overweight as a frequent, potentially long-term consequence of ARFID [31, 62, 63], contrasting most previous publications focusing on ARFID associated underweight status [3, 13, 64, 65]. Future research is needed to investigate the extent to which ARFID may be causally related to obesity.

Psychological impairment was reported with similar frequency (43.8%) to physical impairment in this sample, including reduced mood and mental capabilities, inadequate coping strategies, social-emotional insecurities, fear of being negatively perceived by others, or eating-related shame. For most participants, the psychological strain resulted from the discrepancy between their limited food variety or insufficient energy intake and the perceived inability to change – reflecting a learned helplessness‑like experience, as seen in individuals with depression [66, 67]. Social experiences, such as criticism, social pressure, or the need of justifying one’s eating behavior from the theme “social responses” – mentioned by 24.0% of the sample as impairing – may amplify the psychological distress. These findings are consistent with earlier studies and case reports of adults with ARFID documenting fear of negative evaluation [68], reduced mental health-related quality of life [30, 69], and experiences of social withdrawal and loneliness [19, 22]. Elevated levels of comorbid mental health conditions in ARFID including depressive symptoms and anxiety [7, 68, 69], suggest that psychological distress does not singularly arise from ARFID-related functional impairment or mental health conditions secondary to ARFID, but may also reflect an elevated vulnerability to mental health conditions.

Another source of impairment resulted from restrictions of everyday life (39.6%) regarding social and work contexts, such as avoidance of social gatherings with friends and colleagues, restrictions in leisure time activities or travel options, and some reporting financial hardship as a consequence to food costs. These restrictions align with the personal impairment previously described in adult case studies [20, 21, 23] and restrictions of personal freedom experienced by *n* = 9 adults with ARFID in a qualitative study [25]. Given that most research predominately utilized generic questionnaires on health-related quality of life to quantify impairment in adults with ARFID [30, 61, 69] these specifications of everyday life restrictions may facilitate a more nuanced understanding of the impact of living with ARFID.

Regarding eating behavior (34.4%) as another predominant cause of impairment, adults reported distress about their limited food variety and quantity, an unhealthy diet, and loss of eating-related pleasure. While reduced anticipatory pleasure has previously been linked to more severe ARFID symptomatology [70] the inability to implement dietary alternatives – leading to an unhealthy diet – highlights a new clinically relevant aspect. In many cases, an unhealthy diet entailed the inability to gain or lose weight to reach a healthy BMI thereby resulting in a negative self-perception. A comparable psychological mechanism characterized by the inability to change behavioral patterns and subsequent negative self-perception has been reported for other mental conditions, including attention-deficit/hyperactivity disorder [71, 72] and depression [73, 74]. However, these conditions typically do not entail the same level of physical consequences as those observed in individuals with ARFID. Importantly, unlike other restrictive eating disorders, such as anorexia nervosa, avoidant-restrictive eating patterns are unintentional and themselves present a source of impairment for individuals with ARFID. Incorporating acceptance-based strategies [75] to deal with one’s own eating behaviors due to longstanding sensory sensitivities and lack of interest in eating may be valuable [76, 77], alongside cognitive-behavioral treatment emphasizing, for example, food exposure [5, 78–80].

Beyond social impairment, impairments in partnership (13.5%), particularly, restrictions in joint activities such as cooking or dining out, and parenthood (8.3%) including the concern about being or becoming a good role model for a healthy diet were reported. While similar domains of impairment have been documented in caregivers of children with ARFID [15–17] the present study is the first to report these impairments in adults. The synopsis of self-reported impairments in adults with ARFID and caregiver-reported family strain implies a bidirectional dynamic of personal impairment and effects on family functioning and partnership quality. Since women and men in Germany become parents for the first time at the ages of 30.4 and 33.3 years, respectively – and even later among those with higher levels of education [81, 82] – and the current sample demonstrated a median age of 27 years and 62.5% attained ≥ 12 years of education – it seems plausible that burdensome themes such as parenthood or partnership may appear more frequently in older samples and are underrepresented in this sample.

The fact that ARFID currently remains largely unrecognized both within health care systems and among the general public – especially in countries using the ICD-11 [54] – may contribute to underdiagnosis and stigmatization [39, 83]. In the present study, experiences with practitioners were reported by 2.1% as the main source of impairment, including traumatizing food withholding strategies and prejudices. Notably, most participants reported never having received specific treatment for ARFID. The dearth of knowledge and uncertainty surrounding the management of ARFID is likely attributable to the current absence of diagnostic and treatment guidelines [84].

The additional presentation-based analyses suggest that the impairment findings should be interpreted in the context of substantial ARFID heterogeneity. Sensory sensitivity/picky eating was highly prevalent in the sample (96.9%) – in line with other population-based data [9, 85, 86] – but it frequently co-occurred with lack of interest/low appetite, fear of aversive consequences, and additional eating-related problems. Thus, the findings are best understood as reflecting impairment and illness trajectories in an adult ARFID sample with highly prevalent sensory sensitivity/picky eating embedded in broader, overlapping presentation patterns. The relatively even distribution of impairment themes across ARFID presentations may partly reflect the predominance of sensory sensitivity/picky eating in this sample, however, it may also indicate that impairment in adults with ARFID is not strongly attributable to presentation type.

Regarding ARFID progression, the average age of ARFID symptom onset in the present study was around the age of four years aligning with Thomas et al. [35] and supporting the suggestion of pronounced chronicity of ARFID symptoms in adults [36, 87]. Despite this overall chronicity, four distinct ARFID progression patterns were identified: improved, deteriorated, consistent, and phasic progressions, underscoring the heterogeneity of ARFID progression. All progression groups reported high, non-significantly different levels of food neophobia and picky eating, consistent with previous evidence that features of sensory sensitivity may be relatively stable over time [88]. Although sensory sensitivity/picky eating was the most prevalent presentation across groups, the deteriorated group showed higher fear of aversive consequences than the improved group, with a medium-sized effect. This finding was accompanied by a later symptom onset and shorter illness duration in the deteriorated group compared with the other groups, while sensory sensitivity was associated with earlier onset and longer duration. Taken together, this pattern may suggest that sensory sensitivity/picky eating often represents an early-emerging and persistent feature of ARFID, whereas fear-based avoidance may be more closely linked to later-emerging or escalating symptom courses, potentially following aversive eating-related experiences. Accordingly, perceived deterioration in adulthood may not necessarily reflect worsening of long-standing sensory sensitivity alone but may also involve the emergence or intensification of fear-based avoidance. This interpretation is consistent with prior evidence indicating that the fear of aversive consequences presentation is common in adult clinical samples and that its onset is often linked to one or more triggering eating-related events, either independently or in the context of an underlying sensory sensitivity predisposition [35, 44, 89]. Clinically, this may be important because previous research suggests that individuals with fear of aversive consequences particularly benefit from cognitive-behavioral interventions [90]. Further longitudinal research is needed to identify predictors and influencing factors of ARFID progression and persistence, including the potential role of fear-based avoidance in symptom deterioration.

Beyond ARFID presentation characteristics, sociodemographic factors may also have contributed to perceived symptom progression. Participants with consistent or improved progressions showed higher educational attainment than those with a deteriorated progression, with medium-sized effects, consistent with findings from other mental disorders [91, 92]. Higher education may be associated with greater health literacy, access to resources, or a greater ability to recognize and report symptom change [93, 94]. Moreover, participants in the improved progression group were older than those in the deteriorated group (31.6 vs. 26.9 years, medium effect), which may reflect more time to develop coping strategies, adapt daily routines, or find supportive environments.

A notable strength of the study is the inclusion of *N* = 97 adults with an interview-based ARFID diagnosis. The qualitative approach employed is particularly well-suited to attain a multifaceted and context-sensitive understanding of impairments and progression patterns [57, 58, 95]. Involving several coders in the data analysis, as well as the iterative further development of the categories and themes in exchange with the entire research team represents a further strength. However, the data for the qualitative analysis were derived from answers in an online questionnaire and not validated via qualitative interviews. Further, the cross-sectional study design imposes limitations on causality and prospective illness trajectories. Regarding ARFID presentations, the findings are best understood as reflecting impairment in an adult ARFID sample with highly prevalent sensory sensitivity/picky eating embedded in broader, overlapping presentation patterns. Therefore, the extent to which these findings generalize to individuals with predominantly fear-based or lack-of-interest/low-appetite presentations, especially in clinical samples, remains unclear. A further limitation concerns the fact that recruitment source was not assessed from the beginning of the study and that this information was not available for a substantial subset of participants. In this context, participants recruited through disorder-related self-help or advocacy channels may differ from those recruited through other routes in terms of illness awareness, perceived impairment, chronicity, or motivation to participate. Therefore, findings should be generalized cautiously to clinical treatment-seeking samples and to samples recruited primarily through self-help groups. Finally, given the western European cultural background, the results might not be transferable to other cultural contexts, as recent research stressed differences in ARFID presentation depending on cultural contexts [96, 97].

## Conclusion

In conclusion, this study highlighted the diversity and the interconnection of different sources of impairment in adults with ARFID comprising impairment in health, everyday life, eating behavior, and burden within personal contexts such as family and partnership, but also experiences with health care professionals. Simultaneously, this study underscored the heterogeneity of ARFID progression patterns including improved, deteriorated, consistent, and phasic symptomatology and identified differences in age, education level, and eating-related fears, while sensory sensitivity was highly prevalent across all progression groups. Clinically, the present results may represent a basis to refine diagnostic tools to assess ARFID-related impairment. At the same time, the consideration of individual therapy goals to improve everyday impairments seems warranted – in addition to clinical goals, such as increasing food intake and variety, correcting nutritional deficiencies, or weight gain. Scientifically, prospective studies on predictors of ARFID progression and remission are necessary to identify early interventions targets and further delineate progression patterns.

## Supplementary information

Below is the link to the electronic supplementary material.

Supplementary Material 1: Supplemental Table S1-Supplemental Table S5

## Data Availability

The data supporting the findings of this study are not public due to ethical and privacy concerns but are available upon reasonable request from the corresponding author (R.S.).
