# Supplementary material for: Avoidant/Restrictive food intake disorder in adults within the community: A mixed-method study on individual impairment and progression
Source: J Eat Disord. 2026 Sep 10;14:218. doi: 10.1186/s40337-026-01765-w (PMC13563864; doi:10.1186/s40337-026-01765-w)
Supplement: Supplementary file 1 [file 40337_2026_1765_MOESM1_ESM.docx]

**Supplementary Material**

**Supplemental Table S1.** Sociodemographic and clinical characteristics of ARFID presentations.

|  | NIAS | | |  | EDE-ARFID module | | | | | |
| --- | --- | --- | --- | --- | --- | --- | --- | --- | --- | --- |
|  | Picky eating (*n*=89) | Appetite (*n*=31) | Fear  (*n*=11) |  | Sensory sensitivity (*n*=94) | Lack of interest  (*n*=39) | Eating-related anxiety  (*n*=47) | Emotional problems  (*n*=17) | Ritualized eating  (*n*=13) | Physical problems  (*n*=13) |
|  | *M (SD)* or  *n* (%) | *M (SD)* or  *n* (%) | *M (SD)* or  *n* (%) |  | *M (SD)* or  *n* (%) | *M (SD)* or  *n* (%) | *M (SD)* or  *n* (%) | *M (SD)* or  *n* (%) | *M (SD)* or  *n* (%) | *M (SD)* or  *n* (%) |
| Age | 29.6 (8.7) | 28.5 (9.3) | 28.5 (7.5) |  | 29.5 (8.7) | 29.4 (8.8) | 29.8 (8.6) | 27.3 (7.5) | 32.5 (10.3) | 31.5 (9.1) |
| Gender, female | 62 (71.3) | 21 (72.4) | 8 (80.0) |  | 65 (71.4) | 27 (73.0) | 30 (65.2) | 13 (76.5) | 9 (81.8) | 8 (72.7) |
| Education, ≥12years | 54 (61.4) | 20 (66.7) | 10 (90.9) |  | 58 (62.4) | 21 (55.3) | 29 (61.7) | 10 (62.5) | 7 (53.8) | 8 (61.5) |
| BMI, kg/m² | 24.2 (7.8) | 19.1 (5.1) | 20.5 (6.3) |  | 23.8 (7.4) | 22.0 (7.0) | 25.2 (8.2) | 21.4 (5.9) | 24.1 (8.8) | 20.5 (5.7) |
| ARFID duration (% of lifetime) | 88.0 (16.1) | 82.5 (26.3) | 73.0 (34.0) |  | 86.7 (18.5) | 82. 7 (25.4) | 84.7 (18.8) | 86.5 (18.5) | 87.2 (14.1) | 87.5 (14.7) |
| Age of symptom onset | 3.3 (3.9) | 4.6 (6.4) | 7.6 (8.8) |  | 3.6 (4.7) | 4.7 (6.4) | 4.4 (5.0) | 3.3 (3.8) | 4.7 (6.3) | 4.6 (6.4) |
| NIAS, 0-15 |  |  |  |  |  |  |  |  |  |  |
| Picky eating | 14.0 (1.4) | 12.5 (3.0) | 11.4 (3.9) |  | 13.6 (2.3) | 13.0 (2.8) | 13.6 (2.3) | 13.4 (2.2) | 13.2 (2.9) | 12.9 (2.9) |
| Appetite | 5.8 (4.7) | 11.9 (1.7) | 11.2 (3.3) |  | 6.0 (4.8) | 9.8 (3.8) | 5.6 (4.9) | 9.2 (4.2) | 8.00 (3.5) | 10.9 (4.2) |
| Fear | 3.0 (4.0) | 5.1 (5.3) | 11.5 (4.3) |  | 2.9 (4.1) | 3.9 (4.8) | 4.8 (4.7) | 4.7 (5.5) | 3.6 (3.8) | 7.2 (6.0) |
| EDE-ARFID Module, 0-6 |  |  |  |  |  |  |  |  |  |  |
| Sensory sensitivity | 4.6 (0.6) | 4.5 (0.6) | 4.3 (0.6) |  | 4.6 (0.6) | 4.7 (0.6) | 4.6 (0.8) | 4.5 (0.5) | 5.0 (0.7) | 4.8 (0.7) |
| Lack of interest | 2.6 (2.0) | 4.1 (1.2) | 3.8 (1.5) |  | 2.6 (2.0) | 4.6 (0.6) | 2.3 (2.0) | 3.9 (1.3) | 4.0 (1.6) | 4.5 (1.0) |
| Eating-related anxiety | 2.6 (1.8) | 2.6 (1.7) | 4.2 (0.9) |  | 2.7 (1.8) | 2.6 (1.7) | 4.3 (0.5) | 2.7 (1.6) | 2.5 (1.7) | 3.2 (2.0) |
| Emotional problems | 1.5 (1.7) | 2.5 (1.6) | 2.7 (1.3) |  | 1.6 (1.6) | 2.3 (1.6) | 1.6 (1.6) | 4.2 (0.4) | 2.1 (1.7) | 2.5 (1.5) |
| Ritualized eating | 1.5 (1.6) | 1.7 (1.5) | 1.6 (1.4) |  | 1.5 (1.6) | 1.8 (1.6) | 1.5 (1.6) | 1.5 (1.6) | 4.4 (0.7) | 2.1 (1.8) |
| Physical problems | 1.0 (1.6) | 2.0 (1.9) | 2.9 (2.1) |  | 1.2 (1.6) | 1.7 (1.8) | 1.3 (1.7) | 1.8 (1.9) | 1.6 (1.9) | 4.2 (0.4) |
| Food neophobia, 10-50 | 42.9 (4.7) | 39.5 (8.6) | 35.4 (7.7) |  | 42.0 (6.6) | 40.9 (7.9) | 42.4 (6.1) | 39.9 (8.7) | 43.0 (8.3) | 37.5 (9.0) |
| Sensory Summary, 0-10 | 6.2 (1.6) | 6.7 (1.6) | 6.6 (1.8) |  | 6.2 (1.6) | 6.7 (1.5) | 6.0 (1.7) | 6.5 (1.7) | 7.8 (1.1) | 7.0 (1.3) |
| Temperament (ATQ), mean scores |  |  |  |  |  |  |  |  |  |  |
| Discomfort 1-6 | 4.1 (1.4) | 4.6 (1.5) | 5.0 (1.4) |  | 4.2 (1.4) | 4.7 (1.3) | 4.0 (1.4) | 4.7 (1.5) | 5.7 (0.9) | 4.8 (1.2) |
| Sociability 1-5 | 3.8 (1.3) | 3.4 (1.3) | 3.7 (0.7) |  | 3.8 (1.3) | 3.5 (1.4) | 3.8 (1.4) | 3.8 (1.2) | 2.5 (1.0) | 2.9 (1.0) |
| Obsessive compulsive behavior (FFOCI), 4-20 |  |  |  |  |  |  |  |  |  |  |
| Inflexibility | 13.6 (3.1) | 13.3 (3.5) | 12.0 (4.1) |  | 13.4 (3.2) | 13.1 (3.7) | 13.3 (3.4) | 12.8 (3.3) | 15.5 (2.9) | 13.2 (3.6) |
| Excessive worry | 15.1 (3.9) | 14.8 (4.1) | 16.1 (3.4) |  | 15.1 (3.9) | 15.0 (3.9) | 15.7 (3.6) | 17.3 (2.8) | 14.3 (4.0) | 15.4 (3.3) |
| Risk aversion | 12.1 (2.2) | 12.2 (2.4) | 11.7 (3.1) |  | 12.0 (2.2) | 12.0 (2.4) | 11.7 (2.5) | 11.9 (2.4) | 13.5 (1.9) | 12.1 (2.1) |
| Perfectionism | 14.1 (3.2) | 14.4 (3.4) | 14.5 (3.1) |  | 13.9 (3.3) | 14.0 (3.7) | 14.1 (3.3) | 13.5 (3.2) | 13.9 (3.5) | 12.9 (3.6) |
| Fastidiousness | 13.3 (3.7) | 13.2 (3.9) | 13.3 (4.3) |  | 13.1 (3.8) | 13.4 (4.0) | 13.3 (3.9) | 13.6 (4.1) | 14.7 (3.8) | 12.6 (4.0) |

*Note.* In line with recommendations by Burton Murray et al. (2021), clinically relevant symptomology was defined using NIAS subscale cut-offs of ≥10 for picky eating and fear, and ≥9 for appetite. Clinically relevant EDE-ARFID module presentations were indicated by scores ≥4 on a 0-6 scale. *Abbreviations:* ARFID, avoidant/restrictive food intake disorder; BMI, body mass index (kg/m²), NIAS, Nine-Item ARFID Screener; EDE-ARFID module, Eating Disorder Examination ARFID Module; ATQ, Adult Temperament Questionnaire; FFOCI, Five-Factor Obsessive-Compulsive Inventory.

^1^ Gender was dichotomized into female and male for analysis.

^2^ Education level was dichotomized in *≥* 12 years of school and < 12 years of school.

**Supplemental Table S2.** EDE-ARFID and NIAS subscale convergence and sensitivity/specificity

| **Presentation** | **EDE-ARFID ≥ 4, *n*** | **NIAS above cut-off, *n*** | **Phi** | **Spearman ρ** | **Sensitivity** | **Specificity** |
| --- | --- | --- | --- | --- | --- | --- |
| Lack of interest/ low appetite | 39/97 | 31/96 | .58 | .696 | 65.8 | 89.7 |
| Sensory sensitivity/ picky eating | 94/97 | 89/97 | .38 | .144 | 93.6 | 66.7 |
| Fear of aversive consequences | 47/97 | 11/97 | .17 | .423 | 17.0 | 94.0 |

*Note.* EDE-ARFID presentation ratings ranged from 0 to 6, ratings ≥4 were considered clinically relevant (Schmidt & Hilbert, 2024). NIAS cut-offs were applied according to Burton Murray et al. (2021). Sensitivity and specificity refer to NIAS cut-off classifications relative to clinically relevant EDE-ARFID presentation ratings. Because ARFID presentations were not mutually exclusive, participants could be represented in more than one presentation domain. *Abbreviations:* ARFID, avoidant/restrictive food intake disorder; NIAS, Nine-Item ARFID Screener; EDE-ARFID module, Eating Disorder Examination ARFID Module.

**Supplemental Table S3.** Example of a qualitative analysis process

| **Full-Text Answer** | **Code** | **Category** | **Theme** |
| --- | --- | --- | --- |
| I would like to eat healthier and to be able to cook together with my partner. | Desire for healthier eating; desire to cook together with partner. | Unhealthy Diet;  Restriction of Activity in Partnership | Eating Behavior;  Partnership |

**Supplemental Table S4.** Sociodemographic information of the phasic progression group and comparison with other progression groups

|  | Phasic  (*n*=7) | Improved  vs.  Phasic | Deteriorated  vs.  Phasic | Consistent  vs.  Phasic | |
| --- | --- | --- | --- | --- | --- |
|  | *M (SD)* or *n* (%) | Effect sizes (*d*/*V*) [95% CI] | | |  |
| Sociodemographics |  |  |  |  | |
| Age, years | 34.14 (11.39) | -0.25 [-1.04, 0.55] | -1.01 [-1.90, -0.10] | -0.70 [-1.55, 0.13] | |
| Gender^1^ |  | 0.04 [0.01; 0.38] | 0.01 [0.01; 0.46] | 0.07 [.001 0.39] | |
| Female | 5 (71.4) |  |  |  | |
| Nationality^2^ |  | 0.21 [0.04; 0.61] | 0.33 [0.23; 0.69] | 0.11 [0.02; 0.53] | |
| German | 6 (85.7) |  |  |  | |
| Education level^3^ |  | 0.16 [0.01; 0.49] | 0.26 [0.02; 0.66] | 0.08 [.01; 0.42] | |
| (≥ 12 school years) | 4 (57.1) |  |  |  | |
| Marital status^4^ |  | 0.12 [0.01; 0.49] | 0.25 [0.02; 0.71] | 0.24 [0.02; 0.67] | |
| Single | 4 (57.1) |  |  |  | |
| Married | 2 (28.6) |  |  |  | |
| Anthropometrics |  |  |  |  | |
| BMI, kg/m² | 23.34 (5.05) | 0.10 [-0.70, 0.89] | 0.21 [-0.65, 1.06] | 0.12 [-0.61, 1.05] | |
| ARFID duration (% of lifetime) | 57.90 (36.02) | 1.80 [0.92, 2.67] | 0.62 [-0.26, 1.48] | 1.85 [0.93, 2.76] | |
| Age of onset (years) | 11.86 (7.86) | -2.34 [-3.26, -1.40] | -0.82 [-1.70, 0.08] | -1.93 [-2.84, -1.00] | |

*Note.* Cohen’s *d* and Cramér’s *V* were used to assess effect sizes in group comparisons, interpreted as small (*d*=0.2; *V* = 0.1), medium (*d*=0.5; *V*=0.3) and large (*d*=0.8; *V*=0.5). 95% percentile confidence intervals for Cramer’s *V* were estimated via bootstrapping (5,000 resamples). *Abbreviations:* ARFID, avoidant/restrictive food intake disorder; BMI, body mass index (kg/m²).

^1^ Gender was dichotomized into *female* and *male* for analysis.

^2^ Nationality was grouped into *German* and *Other* due to low frequencies in individual categories.

^3^ Education level was dichotomized in *≥ 12 years of school* and *< 12 years of school*.

^4^ Marital status was dichotomized into *single* and *married*.

**Supplemental Table S5.** Clinical characteristics of the phasic progression group and comparisons with other progression groups

|  | Phasic  (*n*=7) | Improved  vs.  Phasic | Deteriorated  vs.  Phasic | Consistent  vs.  Phasic |
| --- | --- | --- | --- | --- |
|  | *M (SD)* or *n* (%) | Effect sizes (*d*/*V*) [95% CI] | | |
| Avoidant/restrictive behavior |  |  |  |  |
| NIAS picky eating, 0-15 | 12.00 (4.16) | 0.75 [-0.07, 1.55] | 0.57 [-0.31, 1.43] | 0.33 [-0.48, 1.14] |
| Above cut-off ≥ 10 | 5 (71.4) | 0.30 [0.04, 0.68] | 0.31 [0.14, 0.21] | 0.21 [0.01, 0.61] |
| NIAS appetite, 0-15 | 8.00 (3.70) | -0.66 [-1.46, 0.15] | -0.39 [-1.25, 0.48] | -0.27 [-1.08, 0.54] |
| Above cut-off ≥ 9 | 3 (42.9) | 0.13 [0.01, 0.45] | 0.18 [0.01, 0.61] | 0.00 [0.00, 0.36] |
| NIAS fear, 0-15 | 6.24 (5.76) | -1.10 [-1.92, -0.28] | -0.32 [-1.18, 0,54] | -0.54 [-1.36, 0.28] |
| Above cut-off ≥ 10 | 3 (42.9) | 0.43 [0.06, 0.80] | 0.34 [0.03, 0.76] | 0.30 [0.02, 0.68] |
| EDE-ARFID module presentations ≥4 |  |  |  |  |
| Sensory sensitivity | 4.43 (0.98) | 0.08 [-0.72, 0.87] | 0.05 [-0.80, 0.90] | 0.13 [-0.68, 93] |
| Eating related anxiety | 3.43 (1.27) | -0.63, [-1.43, 0.18] | 0.01 [-0.75, 0.95] | -0.51 [-1.32, 0.30] |
| Lack of interest | 4.00 (2.24) | -0.83 [-1.64, -0.02] | -0.66 [-1.52, 0.22] | -0.65 [-1.46, 0.17] |
| Emotional problems | 2.00 (1.29) | -0.23 [-1.02, 0.57] | -0.42 [-1.28, 0.44] | -0.46 [-1.27, 0.36] |
| Ritualized eating behavior | 2.86 (1.35) | -0.92 [-1.73, -0.10] | -1.59 [-2.55, -0.61] | -0.61 [-1.43, 0.21] |
| Physical problems | 0.95 (1.52) | -0.65 [-1.47, 0.16] | -0.64 [-1.50, 0.24] | -0.41 [-1.21, 0.41] |
| Food neophobia, 10-50 | 40.14 (7.06) | 0.29 [-0.51, 11] | 0.49 [-0.38, 1.34] | 0.18 [-0.62, 0.99] |
| Sensory sensitivities, 0-10 |  |  |  |  |
| Sensory summary, mean Score | 6.19 (0.86) | 0.08 [-0.71, 0.87] | -0.20 [-1.05, 0.66] | 0.04 [-0.77, 0.84] |
| Temperament (ATQ), mean scores |  |  |  |  |
| Discomfort 1-6 | 4.65 (1.08) | -0.27 [-10.6, 0.53] | -0.60 [-1.46, 0.27] | -0.24 [-1.04, 0.57] |
| Sociability 1-5 | 3.26 (1.20) | 0.46 [-0.34, 13] | 0.31 [-0.55, 1.16] | 0.30 [-0.51, 1.11] |
| Obsessive compulsive behavior (FFOCI), 4-20 |  |  |  |  |
| Inflexibility | 13.14 (4.06) | 0.15 [-0.64, 0.94] | -0.16 [-1.01, 0.69] | 0.27 [-0.54, 1.08] |
| Excessive worry | 11.71 (2.56) | 0.91 [0.91, 1.72] | 1.27 [0.33, 2.19] | 0.78 [-0.05, 1.60] |
| Risk aversion | 14.43 (3.50) | 0.05 [-0.75, 0.84] | -0.03 [-0.88, 0.82] | 0.16 [-0.65, 0.96] |
| Perfectionism | 12.57 (3.41) | 0.48 [-0.32, 1.27] | -0.36 [-0.50, 1,21] | 0.48 [-0.34, 1.29] |
| Fastidiousness | 11.29 (2.56) | 0.56 [-0.24, 1.36] | 0.33 [-0.53, 1.19] | 0.64 [-0.19, 1.45] |

*Note****.*** Cohen’s *d* and Cramér’s *V* were used to assess effect sizes in group comparisons, interpreted as small (*d* = 0.2; *V* = 0.1), medium (*d* = 0.5; *V* = 0.3) and large (*d* = 0.8; *V* = 0.5). 95% percentile confidence intervals for Cramer’s *V* were estimated via bootstrapping (5,000 resamples). For Cohen’s *d*, Hedges correction was applied due to different group sizes. *Abbreviations:* NIAS, Nine-Item ARFID Screener; EDE-ARFID module, Eating Disorder Examination ARFID Module; ATQ, Adult Temperament Questionnaire; FFOCI, Five-Factor Obsessive-Compulsive Inventory.
